# MiRNAs Regulating Oxidative Stress: A Correlation with Doppler Sonography of Uteroplacental Complex and Clinical State Assessments of Newborns in Fetal Growth Restriction

**DOI:** 10.3390/jcm9103227

**Published:** 2020-10-08

**Authors:** Vladislava Gusar, Mariya Ganichkina, Vitaliy Chagovets, Nataliya Kan, Gennadiy Sukhikh

**Affiliations:** 1 Laboratory of Applied Transcriptomics, Federal State Budget Institution “National Medical Research Center for Obstetrics, Gynecology and Perinatology named after Academician V.I. Kulakov of the Ministry of Healthcare of the Russian Federation”, Oparin str. 4, 117997 Moscow, Russia; 2 Obstetric Physiological Department, Federal State Budget Institution “National Medical Research Center for Obstetrics, Gynecology and Perinatology named after Academician V.I. Kulakov of the Ministry of Healthcare of the Russian Federation”, Oparin str. 4, 117997 Moscow, Russia; mariaganichkina@yandex.ru; 3 Laboratory of Proteomics and Metabolomics of Human Reproduction, Federal State Budget Institution “National Medical Research Center for Obstetrics, Gynecology and Perinatology named after Academician V.I. Kulakov of the Ministry of Healthcare of the Russian Federation”, Oparin str. 4, 117997 Moscow, Russia; v_chagovets@oparina4.ru; 4 Department for Obstetrics and Gynecology, Professional Education Department, Federal State Budget Institution “National Medical Research Center for Obstetrics, Gynecology and/Perinatology named after Academician V.I. Kulakov of the Ministry of Healthcare of the Russian Federation”, Oparin str. 4, 117997 Moscow, Russia; kan-med@mail.ru; 5 Federal State Budget Institution “National Medical Research Center for Obstetrics, Gynecology and Perinatology named after Academician V.I. Kulakov of the Ministry of Healthcare of the Russian Federation”, Oparin str. 4, 117997 Moscow, Russia; g_sukhikh@oparina4.ru; 6 Department of Obstetrics, Gynecology, Perinatology and Reproductive Medicine, Institute of Professional Education, Federal State Autonomous Educational Institution of Higher Education I.M. Sechenov First Moscow State Medical University of the Ministry of Health of the Russian Federation (Sechenov University), Bolshaya Pirogovskaya str., 2, 119991 Moscow, Russia

**Keywords:** microRNA/miRNA, Fetal Growth Restriction/FGR, oxidative stress, vascular dysfunction, umbilical cord blood, Doppler, pulsatility index, fetal hemodynamics

## Abstract

Overproduction of reactive oxygen species (ROS) and, as a result, uncontrolled oxidative stress (OS) can play a central role in disorders of fetal hemodynamics and subsequent development of adverse perinatal outcomes in newborns with fetal growth restriction (FGR). Given the epigenetic nature of such disorders, the aim of our study was to evaluate the expression of miRNAs associated with OS and endothelial dysfunction (miR-27a-3p, miR-30b-5p, miR-125b-5p, miR-221-3p, miR-451a and miR-574-3p) in umbilical cord blood using real-time quantitative RT-PCR. ΜiRNA expression was evaluated in patients with FGR delivery before (*n* = 9 pregnant) and after 34 weeks of gestation (*n* = 13 pregnant), and the control groups corresponding to the main groups by gestational age (13 pregnant women in each group, respectively). A significant increase in miR-451a expression was detected in late-onset FGR and correlations with fetoplacental and cerebral circulation were established (increase of resistance in the umbilical artery (pulsatility index, PI UA (umbilical artery): r = −0.59, *p* = 0.001) and a decrease in cerebral blood flow (CPR: r = 0.48, *p* = 0.009)). The change in miR-125b-5p expression in the placenta is associated with reduced Doppler of cerebral hemodynamics (CPR: r = 0.73, *p* = 0.003; PI MCA (middle cerebral artery): r = 0.79, *p* = 0.0007), and newborn weight (r = 0.56, *p* = 0.04) in early-onset FGR. In addition, significant changes in miR-125b-5p and miR-451a expression in umbilical cord blood plasma were found in newborns with neonatal respiratory distress syndrome (NRDS) (in early-onset FGR) and very low birth weight (VLBW) (in late-onset FGR). A number of key signaling pathways have been identified in which the regulation of the studied miRNAs is involved, including angiogenesis, neurotrophin signaling pathway and oxidative stress response. In general, our study showed that changes of the redox homeostasis in the mother-placenta-fetus system in FGR and subsequent perinatal outcomes may be due to differential expression of oxidative stress-associated miRNAs.

## 1. Introduction

Among the common obstetric causes of preterm birth and intra-natal asphyxia, as well as the leading causes of perinatal morbidity and mortality, the second highest is fetal growth restriction (FGR). FGR affecting around 5–10% of pregnancies. [1]. This condition occurs when the fetus does not reach its genetically programmed intrauterine potential for growth and development, due to placental dysfunction. In clinical practice, FGR is defined as an estimated fetal weight below the 10th percentile for gestational age (ACOG) [2]. Fetuses with intrauterine growth restriction are at risk of obstetric and neonatal complications, and, at an older age, the development of cardiometabolic diseases [3,4]. The multifactorial etiology underlying FGR suggests that this condition may be the result of both endogenous factors affecting the growth and development of the fetus (fetal factor, including genetic and chromosomal abnormalities and congenital metabolic disorders), impaired placentation and implantation (placental factor) and placental dysfunction (maternal factor), as a result of which the transmission of nutrients and oxygen from the mother to the fetus is impaired (i.e., FGR of vascular origin) [1]. A decrease in uteroplacental perfusion due to increased vascular resistance leads to ischemic reperfusion injury and/or hyperoxia/reoxygenation, the production of excessive amounts of reactive oxygen species (ROS) and uncontrolled oxidative stress (OS) [5,6]. The latter can play a central role in programming the long-term consequences of the development of cardiovascular diseases in the fetus: an increase of superoxide and nitric oxide production with the formation of prooxidant peroxy-nitrite under OS leads to vascular dysfunction [7].

In current clinical practice, for the diagnosis of diseases associated with pathology of the placenta, Doppler sonography and cardiotocography of the fetus are successfully used. Doppler follow-up of the umbilical artery (UA) has a major importance in early-onset FGR. Absence or reverse end diastolic blood flow can indicate a significant decrease in blood flow and severe fetal deterioration. A decrease in the Doppler sonography indices of the middle cerebral artery (MCA) and cerebral placental ratio (CPR) in combination with normal or minimal blood flow disorders in the uterine artery is recorded in patients with late-onset FGR [1,8]. There is also data reflecting the relationship of OS markers in the umbilical cord blood of the fetus with growth restriction and abnormal blood flow in the umbilical artery, via Doppler sonography [9].

The role of microRNAs (miRNAs) as epigenetic modulators of a significant number of biological processes, including those associated with the regulation of the normal development of the placenta and its dysfunction, has been actively studied recently. In this regard, entire classes of functionally specific miRNAs have been identified, in particular, trophomiRs [10], hypoxamiRs [11], and oxidative stress-associated miRNAs [12]. It should be noted that a number of miRNAs previously described relating to gestational complications were investigated in connection with hypoxia and OS in experimental models [13]. Some studies focused on the identification of differentially expressed miRNA patterns in the umbilical cord blood of a newborn, and their comparison with the placental mRNAs expression profile, to search for prognostic and diagnostic markers of gestational pathology [14,15]. However, data relating to the miRNA-dependent regulation of redox homeostasis in the context of pregnancy complications associated with the imbalance of the pro- and antioxidant systems are scarce [12,16]. In addition, given the long-term consequences associated with vascular dysfunction in fetuses with growth restriction, the search for associations between oxidative stress-associated miRNAs and Doppler ultra-sonography of uteroplacental and fetal-placental blood flows is becoming relevant. It should be noted that in our previous studies evaluating the miRNA expression profile associated with OS and placental vascular dysfunction in pregnant women with FGR, the differential expression of miR-125b-5p, miR-221-3p, miR-451a, and miR-574-3p was determined in the placental tissue of pregnant women [17]. In this connection, it was of interest to us in this work to focus on the evaluation of the oxidative stress-associated miRNAs expression in umbilical cord blood plasma, as well as to establish a relationship between fetus hemodynamics with growth restriction and perinatal outcomes.

## 2. Materials and Methods

### 2.1. Study Design and Patient Cohort

**Figure 1:**
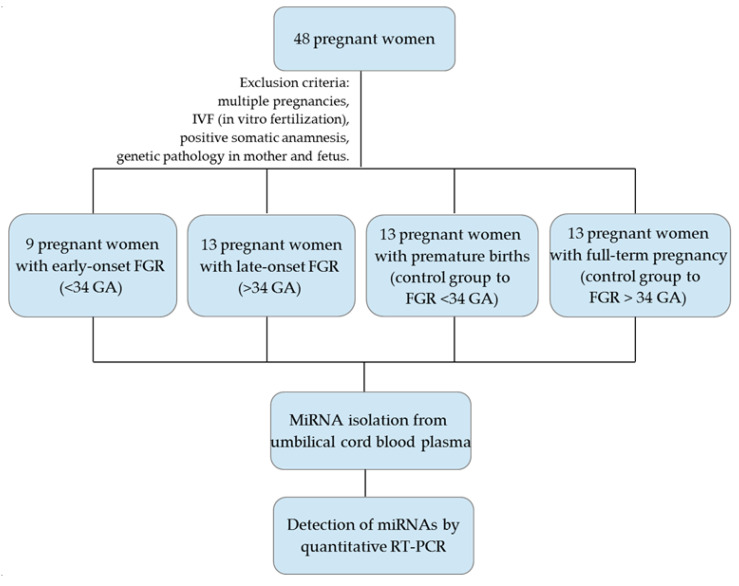
Flowchart of the study population.

The study included pregnant women who were clinically observed at the V. I. Kulakov National Medical Research Center for Obstetrics, Perinatology and Gynecology, Ministry of Healthcare of the Russian Federation. In total, 48 patients of reproductive age were divided into 4 groups: (1) in the group with early-onset FGR pregnant women were included whose delivery was performed before 34 weeks of gestation (*n* = 9; 30.5 ± 1.9 weeks); (2) in the group with late-onset FGR pregnant women were included with delivery after 34 weeks of gestation (*n* = 13; 37 weeks (36.3–38.3 weeks)); and the control groups corresponded to the main groups by gestational age; ((3) 13 pregnant women with premature births up to 34 weeks (31.5 ± 1.9 weeks); (4) 13 pregnant women with full-term (38.2 weeks (36.4–38.5 weeks)) physiological pregnancy) (Figure 1). The exclusion criteria were: pregnant women with multiple pregnancies and IVF (in vitro fertilization), specific family history, maternal and fetal genetic pathology.

### 2.2. Clinical Data, Ultrasound and Doppler Sonography Measurement

FGR was established based on the following criteria: the estimated fetal weight below the 3rd percentile for gestational age, or the abdominal circumference less than the 3rd percentile, or presence null and inversum diastolic blood flow in the umbilical arteries, or abdominal circumference and estimated fetal weight below 10th percentile combined with PI (pulsatility index) in uterine or umbilical arteries greater than 95th percentile. In addition, amniotic fluid deficiency (oligohydramnion and anhydramnion) in pregnant women was determined using ultrasonic diagnostic criteria of oligohydramnios, namely: single deepest vertical pocket (SDVP) < 2 cm, amniotic fluid index (AFI) ≤ 5 cm.

The data of clinical, ultrasound, and Doppler studies are presented in Table 1.

Blood flow in the uterine, middle cerebral and umbilical arteries of the fetus was determined using a Doppler ultra-sonograph (Voluson E10, GE Healthcare Technologies, Milwaukee, WI, USA). When evaluating the velocity curves of blood flow, the calculation of the pulsatility index (PI) was used. To assess the state of the fetus, cardiotocography (CTG) was performed using the automated antenatal monitor AAM-04 (UNICOS, Moscow, Russia) in pregnant women with gestational age over 33 weeks. The automatic calculation program is based on a multi-criteria mathematical analysis of cardiotocography proposed by professor V. Demidov [20]. The study is carried out for 30−60 min, during which the following indicators are monitored: the integral indicator of the fetal condition (IFC), the curve of the fetal heart rate (acceleration and deceleration of the heart rhythm), its motor activity (the number of movements) with correction for sleep cycle, the number of contractions of the uterus, and the presence of its hyper-tonus. The values of IFC are calculated by the formula: IFC = 155 × 10 − 4 (E tcp) + 87 × 10 − 7 × (E hma) − 2 − 64 × 10 − 4 × (E hma) + 0.33/Max hma/cp + 0.05, where E tcp is the total duration of a stable rhythm; E hma is the total amplitude of slow accelerations; and Max hma/cp is the ratio of the amplitude of maximum accelerations to the maximum time of a stable rhythm. Values of IFC are categorized as follows: less than 1, normal state of the fetus; 1.01–2.0, initial impairment of the fetus; 2.01–3.0, severe impairment of the fetus; more than 3, critical state of the fetus.

The delivery of the pregnant women was performed by cesarean. The caesarean in pregnant women with early-onset FGR was performed for the following indications: fetal distress according to Doppler and CTG, severe pre-eclampsia, placental abruption and breech position. The main indications for cesarean in pregnant women with late-onset FGR were: FGR in combination with severe pre-eclampsia, scar after previous cesarean. and a burdened obstetric and gynecological anamnesis. The operative delivery in the control groups was performed due to breech position and uterine scar after previous cesarean.

All studies were carried out with informed consent of patients in accordance with the Helsinki Declaration and were approved by the Commission of Biomedical Ethics (#13, 10.12.2015) at the V. I. Kulakov National Medical Research Center for Obstetrics, Perinatology and Gynecology, Ministry of Healthcare of the Russian Federation.

### 2.3. Clinical State Assessment of Newborns and Neonatal Outcomes

The state of the newborns was evaluated in accordance with accepted criteria, which included: birth weight, Apgar score, stature–weight values, measurement of lactate, acid-base balance, and blood gases. The estimation of fetal weight centiles is given in accordance with INTERGROWTH-21st and the Fenton growth chart. A neuro-sonographic study was performed. A number of clinical characteristics of the newborns are presented in Table 2.

### 2.4. Blood Collection and miRNA Isolation from Umbilical Cord Blood Plasma

Samples of umbilical cord blood plasma, taken during the cesarean from the umbilical vessels (predominantly artery vessel and, immediately after ligation, closer to the placental end) in pregnant women with early -and late-onset FGR and control groups were used for the experimental study. Blood samples were collected into VACUETTE^®^ tubes containing EDTA (BectonDickinson Canada, Mississauga, ON, Canada). The samples were prepared according to the following protocol: whole blood was centrifuged at 300× *g*, 4 °C for 20 min, and then the supernatant was centrifuged at 14,000× *g* for 10 min. For the analysis 200 μL of the prepared plasma was used, and 5.6 × 10^8^ copies of cel-miR-39 (miScript Primer Assay, Qiagen, Hilden, Germany) were added to a plasma sample as an internal control of the efficiency of isolation and subsequent cDNA synthesis for quantitative RT-PCR. Then, miRNA was isolated by using an miRNeasy Serum/Plasma kit (Qiagen). The extraction stages were carried out at the automatic station (QIAcube) in accordance with the protocols of the QIAGEN manufacturing company.

### 2.5. Real-Time Quantitative RT-PCR

For the reverse transcription reaction the following RNA-specific sense primers were used: hsa-miR-27a-3p MIMAT0000084 (5′-TTCACAGTGGCTAAGTTCCGC, Tm = 58.7 °C), hsa-miR-30b-5p MIMAT0000420 (5′-TGTAAACATCCTACACTCAGCT, Tm = 54.6 °C), hsa-miR-125b-5p MIMAT0000423 (5′-TCCCTGAGACCCTAACTTGTGA, Tm = 59.5 °C), hsa-miR-221-3p MIMAT0000278 (5′-AGCTACATTGTCTGCTGGGTTTC, Tm = 59.5 °C), hsa-miR-451a MIMAT0001631 (5′-AAACCGTTACCATTACTGAGTT, Tm = 59.5 °C), hsa-miR-574-3p MIMAT0003239 (5′-CACGCTCATGCACACACCCACA, Tm = 59.5 °C), cel-miR-39, Tm = 55 °C. This reaction was performed using a miScript II RT Kit (Qiagen). To determine the level of miRNA expression in the cord blood plasma, quantitative PCR was performed with a miScript SYBR Green PCR Kit (Qiagen) using a StepOnePlus device (Applied Biosystems, Foster City, CA, USA). The stages were carried out in accordance with the protocols of the QIAGEN manufacturing company. The threshold level of expression was Ct ≤ 36. The level of miRNA expression was determined by the 2^−ΔΔCT^ method [23], using cel-miR-39 miScript Primer Assay (Qiagen) as the reference RNA.

### 2.6. Statistical Data Analysis

The statistical significance of the difference between the levels of miRNA expression in the groups under study was assessed by the Wilcox and Mann–Whitney methods using scripts written in the R language (https://www.R-project.org/). The normality of clinical parameter distribution was evaluated by the Shapiro–Wilk test. Statistical analysis was performed using the Student’s t-test with a normal distribution of the parameter and using the Mann–Whitney test when the distribution did not correspond to the law of normal distribution. To describe quantitative data having a normal distribution, the mean value (M) and standard deviation (SD) in the M ± SD format were used. In case of non-normal distribution, the parameter was described as the median (Me) and quartiles Q1, Q3 in the format Me (Q1–Q3). The Spearman nonparametric rank correlation method was used to evaluate the relationship between the expression level of studied mRNA and clinical parameters. Normalized values of the uterine, umbilical and middle cerebral arteries’ pulsatility indexes (*PI*(*n*)) were used to estimate the difference between the groups of pregnant women. The *PI*(*n*) were calculated according to the following equations:

(1) $$PI\;UtA\left(n\right)=\frac{PI\left(UtA\right)-UtA\left(5\right)}{UtA\left(95\right)-UtA\left(5\right)}*2-1;$$

(2) $$PI\;UA\left(n\right)=\frac{PI\left(UA\right)-UtA\left(5\right)}{UA\left(95\right)-UA\left(5\right)}*2-1;$$

(3) $$PI\;MCA\left(n\right)=\frac{PI\left(MCA\right)-MCA\left(5\right)}{MCA\left(95\right)-MCA\left(5\right)}*2-1,$$

where, *PI* (*UA*, *UtA*, *MCA*) are the values of the pulsatility indexes of the uterine, umbilical and middle cerebral arteries, respectively; *UtA*(5), *UA*(5) and *MCA*(5) are the reference values for the 5th percentiles according to gestational age; and *UtA*(95), *UA*(95) and *MCA*(95) are the reference values for the 95th percentiles according to gestational age [18,19].

## 3. Results

### 3.1. Evaluation of miRNAs Regulating Oxidative Stress Expression in Umbilical Cord Blood Plasma

**Figure 2:**
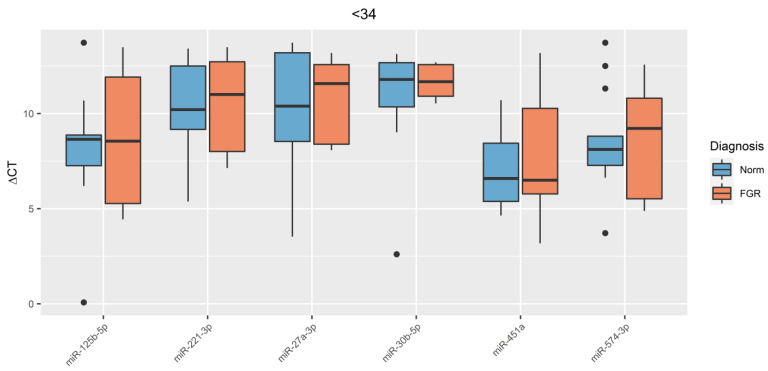
Comparative analysis of expression levels for miR-27a-3p, miR-30b-5p, miR-125b-5p, miR-221-3p, miR-451a, and miR-574-3p in umbilical cord blood plasma in pregnant women with early-onset FGR (FGR < 34). The box diagram shows the medians of ΔCt values, the first and third quartiles, and the edges of the statistically significant sample, and the dots denote the emissions. The lower the value of ΔCt, the higher the level of expression of studied miRNA.

In plasma samples of umbilical cord blood of newborns, miRNA expression (miR-125b-5p, miR-221-3p, miR-451a, and miR-574-3p), previously studied by us in the placenta tissue of pregnant women with FGR, was evaluated [17]. In addition, the expression levels of miR-30b-5p, which regulates the antioxidant enzyme catalase (CAT) and miR-27a-3p, which regulates NRF2 (nuclear transcription factor) and is an initiator of antioxidant gene transcription, were evaluated. The analysis showed that in the umbilical cord blood plasma samples in pregnant women with early-onset FGR (FGR < 34), the expression levels (ΔCt) of studied miRNAs were not statistically significant (*p* > 0.05). (Figure 2).

**Figure 3:**
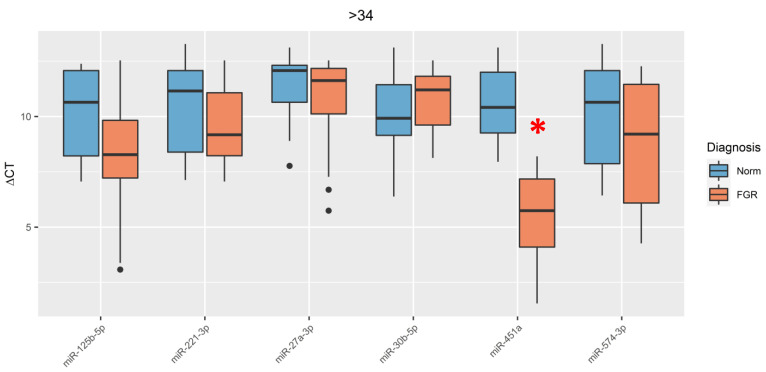
Comparative analysis of expression levels for miR-27a-3p, miR-30b-5p, miR-125b-5p, miR-221-3p, miR-451a, and miR-574-3p in umbilical cord blood plasma in pregnant women with late-onset FGR (FGR > 34). An asterisk marks significant differences in the level of miRNA expression. The box diagram shows the medians of ΔCt values, the first and third quartiles, and the edges of the statistically significant sample, and the dots denote the emissions. The lower the value of ΔCt, the higher the level of expression of studied miRNA.

In pregnant women with late-onset FGR (FGR > 34), only the expression level of miR-451a (5.75; 4.1–7.18) was significantly (*p* < 0.001) increased relative to the control group (Norm) (10.41; 9.26–12) (Figure 3).

### 3.2. Correlation of miRNA Expression in Umbilical Cord Blood with Doppler Measurement of Pregnant Women and Clinical State Assessment of Newborns with FGR

To assess the relationship between the expression level of studied miRNAs and clinical parameters, a correlation analysis was carried out using the Spearman non-parametric rank correlation method in pregnant women and newborns with FGR (Table 3).

No significant correlation between miRNA expression level, clinical data and Doppler studies of pregnant women with early-onset FGR has not been established.

The assessment of the Doppler measurement of the uteroplacental complex established negative correlations with the pulsatility index (PI) value of the umbilical artery, both with ΔCt miR-125b-5p (r = −0.54, *p* = 0.03) and ΔCt miR-451a (r = −0.59, *p* = 0.001) in late-onset FGR. Herewith, the average PI value of the umbilical artery in pregnant women with FGR was 0.75 (0.19–0.96), and in the comparison group, 0 (−0.12–0.19; *p* = 0.008), respectively (see Table 1). In addition, ΔCt miR-451a was negatively correlated with a parameter of fetal condition state (r = −0.38, *p* = 0.04). The lower the value of ΔCt, the higher the level of expression of studied miRNA. Consequently, with an increase in the expression level of miR-451a, the PI of the umbilical artery and IFC are increased. Herewith, ΔCt miR-451a positively correlates with the CPR (r = 0.48, *p* = 0.009): the average CPR in pregnant women with late-onset FGR is 1.45 (1–2.08), and in the control group is 2.11 (2.08–2.21; *p* = 0.02). Note the CPR is within normal limits is >1 (see Table 1). In addition to the established correlations in late-onset FGR, differences in the expression levels of miR-125b-5p (*p* = 0.02) and miR-451a (*p* < 0.001) were found in the group of pregnant women with impaired fetoplacental and uteroplacental blood flow.

It is interesting to note that in late-onset FGR the expression level of studied miRNAs is negatively correlated with the indicators of the blood coagulation system of pregnant women, in particular the platelet (r = −0.51, *p* = 0.005) and the prothrombin index (r = −0.46, *p* = 0.01).

The analysis of the correlation with the clinical assessment of the newborn’s state found that the birth weight positively correlates with ΔCt miR-125b-5p value (r = 0.41, *p* = 0.03). In addition, a search was conducted for the relationships between ΔCt values of studied miRNAs, parameters of acid-base balance and blood gases of newborns. However, no significant correlations were found.

Taking into account the adverse perinatal outcomes in newborns with early- and late-onset fetal growth restriction, we found it interesting to assess the differences in the level of miRNA expression in these groups. The expression of miR-125b-5p (*p* = 0.02) and miR-451a (*p* = 0.05) significantly changes in umbilical cord blood plasma in newborns with neonatal respiratory distress syndrome (NRDS) in early-onset FGR. In late-onset FGR, there are differences in the expression level of miR-125b-5p (*p* = 0.03) and miR-451a (*p* = 0.02) in newborns with very low birth weight (VLBW).

### 3.3. Correlation of miRNA Expression in Placenta with Doppler Measurement of Pregnant Women and Clinical State Assessment of Newborns with FGR

Our previous studies evaluating miRNA expression in placental tissue of pregnant women with FGR showed an increase in miR-125b-5p expression level in early-onset FGR and a decrease in miR-451a expression level in pregnant women with early- and late-onset FGR. Given these results, we undertook the correlation analysis in this research (Table 4).

An analysis of the relationship between miR-125b-5p expression level and clinical data allowed us to establish correlations with ΔCt miR-125b-5p as follows: PI MCA (r = 0.79; *p* = 0.0007), CPR (r = 0.73; *p* = 0.003), estimated fetal weight (EFW) (ultrasonography) (r = 0.54; *p* = 0.05) and weight of newborn (r = 0.56; *p* = 0.04). Given the positive direction of correlations, an increase in miR-125b-5p expression in early-onset FGR can contribute to a decrease in Doppler indices and corresponding changes in the cerebral hemodynamics of the fetus. Moreover, ΔCt miR-451a was correlated with the PI uterine artery (r = 0.62; *p* = 0.01), indicating that the reduced miR-451a expression in the placenta is associated with an increase in pulsatility. It should be noted that ΔCt miR-451a was also correlated with the Apgar score in the first minute (r = −0.57; *p* = 0.01) in late-onset FGR.

### 3.4. Correlation of Doppler Measurement of Pregnant Women with Clinical State Assessment of Newborns with FGR

Given the dependence of reduced uteroplacental perfusion due to increased vascular resistance during the hemodynamic state of the fetus during pregnancy, a correlation analysis was performed between the corresponding parameters (Table 5).

The analysis made it possible to establish negative correlations between IFC, birth weight, and platelet of newborns with FGR. Herewith, the Apgar value of 1 min was inversely correlated with IFC (r = −0.59; *p* = 0.004), PI of uterine artery (r = −0.54; *p* = 0.01) and umbilical arteria (r = −0.44; *p* = 0.04) only in early-onset FGR, indicating the severity of identified disorders. In addition, a positive relationship was established between the Apgar value of 1 min and the CPR (r = 0.55; *p* = 0.01) in early-onset FGR. The values of CPR in this group of pregnant women were reduced (0.94 ± 0.16) relative to the control group (2.33 ± 0.44; *p* <0.001). Furthermore, the Apgar indicator of 5 min was negatively correlated with PI of the umbilical artery in early- (r = −0.42; *p* = 0.05) and late-onset (r = −0.39; *p* = 0.04) FGR. It should be noted that both indicators of Apgar score were positively correlated with EFW, the values of which were reduced in FGR.

Reduced indicators of birth weight were negatively correlated with increased PI of uterine arteries: in early- (r = −0.63; *p* = 0.002) and late-onset (r = −0.38; *p* = 0.05) FGR. Negative correlation with PI of umbilical artery (r = −0.67; *p* = 0.0001) was observed in late-onset FGR. It is interesting to note the correlation between the indicators of fetal state, increased pulsatility indexes of placenta vessels and thrombocytopenia of newborns; furthermore, platelet were correlated with CPR (r = 0.68; *p* = 0.0001) in early-onset FGR and with PI MCA (r = −0.39; *p* = 0.04) - in late-onset FGR.

## 4. Discussion

Obviously, the production of ROS and OS play an important role both in maintaining the physiological state of the placenta and in cases of impaired placental blood flow [24]. The involvement of OS in vascular remodeling, and associated changes in the structure and function of the vascular endothelium, are identified as key factors in the development of cardiovascular diseases, which are subsequently observed in children with FGR [4,25]. Potential programming mechanisms for such long-term effects are being actively explored. Their epigenetic nature is assumed [25,26]. In this regard, the expression of miRNAs associated with OS and endothelial vascular dysfunction in umbilical cord blood plasma of newborns with FGR was evaluated. It should be noted that the expression of these miRNAs (miR-125b-5p, miR-221-3p, miR-451a, and miR-574-3p) significantly changed in placental tissue of pregnant women with early- and late-onset FGR [17]. However, statistically significant differences were detected in umbilical cord blood plasma only in miR-451a expression levels in late-onset FGR. Previously, in studies by Hromadnikova et al. evaluating miRNA expression between groups of pregnant women with gestational hypertension, preeclampsia, and FGR, a decrease in the expression of miR-574-3p was revealed in cord blood only with early-onset severe preeclampsia. In addition, the expression level of miR-221-3p was negatively correlated with PI of umbilical artery (r = −0.390, *p* = 0.017) in FGR [14].

Most non-invasive studies of the function of placenta vessels and the umbilical cord are based on dopplerography [8,27]. Previously, in the study of Maisonneuve E. et al., significant correlations were found of an increased concentration of oxidized low-density lipoproteins in umbilical cord blood of newborns with FGR with abnormal umbilical cord dopplerography [9]. In a study by Hracsko Z. et al., a connection with the oxidative status of newborns with FGR was shown [28]. Expecting that the changes in placental microcirculation in FGR may reflect the relationship between the expression of oxidative stress-associated miRNAs in the umbilical cord vessels and Doppler sonography of the uteroplacental and fetal-placental complex, we conducted an appropriate study. The significant negative correlations of ΔCt miR-451a with PI of the umbilical artery and the fetal state condition index (IFC CTG) in late-onset FGR were established. Therefore, with an increase in miR-451a expression level, these parameters increase. The IFC range indicating normal development of the fetus is 0–1.0 [20]. In 6 out of 13 pregnant women in late-onset FGR, IFC values indicated initial (1.1–2.0) and pronounced (2.1–3.0) disorders of the fetus state.

The ratio between the PI of cerebral and that of fetoplacental circulation, known as the cerebroplacental ratio (CPR) (i.e., PI MCA/PI UA), is one of the parameters that reflect the distribution of cardiac output in favor of cerebral blood flow and has the greatest accuracy in predicting perinatal outcomes [29]. Values of CPR < 1 indicate a redistribution of fetal blood flow to the brain in response to a decrease in pO2. In late-onset FGR, the degree of tolerance to hypoxia is low in contrast to early-onset FGR and therefore, the fetus cannot be under conditions of oxygen deprivation for a long time [30]. The value of ΔCt miR-451a was positively correlated with CPR, therefore an increase in the expression level of this miRNA in umbilical cord blood may be associated with impaired cerebral hemodynamics in the fetus. It is interesting to note that, in placental tissue, we found an association between miR-451a expression and an increase in PI of the uterine artery in early-onset FGR, while in late-onset FGR, ΔCt miR-451a was negatively correlated with the Apgar score at the first minute; that is, the detected correlations indicated dysfunctional blood flows in the mother-placenta-fetus system. Significant differences in the expression levels of miR-451a associated with impaired fetoplacental and uteroplacental blood flow were also revealed in late-onset FGR.

In antenatal adaptation to an environment with a low oxygen content, a decrease in cerebral and coronary vascular resistance is observed [31]. A decrease in PI MCA may indicate delayed fetal asphyxia [32]. In this regard, we found significant correlations of ΔCt miR-125b-5 in placental tissue with PI MCA, CPR, EFW (ultrasonography) and newborn weight in early-onset FGR. In umbilical cord blood the expression value of this miRNA was correlated with PI of the umbilical artery, and changes in its expression were significant in impaired fetoplacental and uteroplacental blood flow in late-onset FGR.

A number of researchers have discovered a connection between the Doppler characteristics of the fetus and its hemodynamic parameters after birth [30,33]. In this regard, a correlation analysis of Doppler measurement in pregnant women with clinical state parameters of newborns with FGR was conducted (see Table 5). The analysis demonstrated the inverse relationship between increased resistance of the placenta and umbilical vessels and decreased fetal cerebral hemodynamics, birth weight, APGAR scores, and newborn thrombocytopenia. According to Baschat et al., a decrease in platelets negatively correlates with Doppler measurement of the umbilical artery, which is associated with their increased consumption against a background of reduced production [34]. A study by Martinelli et al. indicated a positive correlation between PI MCA and platelets associated with hypoxemia [35]. It should be noted that in our work platelets and the prothrombin index of pregnant women were correlated with ΔCt values of miR-125b-5p and miR-451a in late-onset FGR. Other authors have previously showed a relationship between the expression level of these miRNAs and platelets of peripheral blood plasma [36,37]. Taking into account the adverse perinatal outcomes in newborns with early- and late-onset growth restriction, we assessed the differences in the expression level of under study miRNAs in these groups. The analysis showed that the expression of miR-125b-5p and miR-451a significantly changes in umbilical cord blood plasma in newborns with NRDS in early-onset FGR. In late-onset FGR, there are differences in the expression level of miR-125b-5p and miR-451a in newborns with VLBW.

A number of studies have proven the effect of exosomes on endothelial function, including vascular tone, interaction between endothelial cells, and angiogenesis [38,39,40]. In the case of placenta-associated pathologies, the release of exosomes from the cells increases, since this process is regulated by changing the oxygen tension and glucose concentration, as well as the pro- and antioxidant balance. In addition, miRNAs, as important mediators in the regulation of intercellular communication, compete in exosomes targeted by endothelial cells. Moreover, miRNAs can suppress or enhance angiogenesis, thus realizing the fetal-maternal signal transmission mechanism leading to vascular maladaptation of the fetus [40]. According to the data of Huang H. et al., miR-125b-5p and miR-451a are the most abundant in exosomes [41]. In connection with the above, we suggest that a change in miR-125b-5p and miR-451a expression in FGR can contribute to reducing the vascularization of placental villi through inhibition of angiogenic and proliferative factors. This leads to an increase in peripheral resistance of the placenta vessels, and consequently an increase in IFC and PI of umbilical and uterine arteries, and a decrease of CPR and PI MCA.

**Figure 4:**
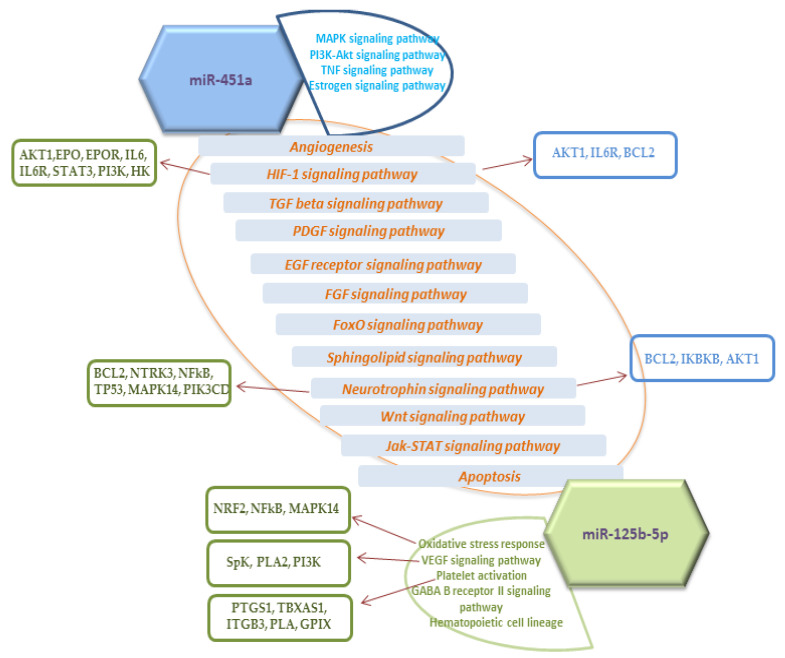
Signaling pathway potentially regulated by studied miRNAs (*p*-value ≤ 0.05). Pathways included within the oval are common to miR-125b-5p and miR-451a. Lists of signaling pathways next to the figures with the name of miRNA are specific to the miRNAs. Target genes regulated by these miRNAs and participating in the corresponding signaling pathways are indicated in blocks with arrows. The color of the blocks corresponds to the color of the figures with the name of the miRNA. Names of signaling pathways: hypoxia’s response via HIF activation (HIF-1 signaling pathway), transforming growth factor beta (TGF-β signaling pathway), platelet-derived growth factor (PDGF signaling pathway), epidermal growth factor receptor (EGF receptor signaling pathway), fibroblast growth factor (FGF signaling pathway), transcription factor FoxO (FoxO signaling pathway), involving sphingolipids (Sphingolipid signaling pathway), regulated by neurotrophins (neurotrophin signaling pathway), group of signal transduction pathways (Wnt signaling pathway), Janus kinase/signal transducers and activators of transcription (JAK/STAT) pathway (Jak-STAT signaling pathway). Names of genes potentially regulated by studied miRNAs (*p*-value ≤ 0.05): AKT1—Serine/Threonine Kinase 1, EPO and EPOR—Erythropoietin and his receptor, IL6 and IL6R—Interleukin 6 and its receptor, STAT3—signal transducer and activator of transcription 3, PI3K—Phosphoinositide 3-kinase, HK—Hexokinase, BCL2—Apoptosis regulator Bcl-2, NTRK3—Neurotrophic Receptor Tyrosine Kinase 3, NFkB—nuclear transcription factor kappa B, TP53—Tumor Protein P53, MAPK14—Mitogen-Activated Protein Kinase 14, PIK3CD—Phosphatidylinositol-4,5-Bisphosphate 3-Kinase Catalytic Subunit Delta, IKBKB—Inhibitor Of Nuclear Factor Kappa B Kinase Subunit Beta, NRF2—Nuclear factor erythroid 2-related factor 2, SpK—Sphingosine kinase, PLA2—Phospholipase A2, PTGS1—Prostaglandin-Endoperoxide Synthase 1, TBXAS1—Thromboxane A synthase 1, ITGB3—Integrin Subunit Beta 3 (Platelet Glycoprotein IIIa), PLA—Plasminogen activator, GPIX—Glycoprotein IX (platelet).

It is known that the physiological and pathological effects of miRNAs secreted by the placenta and circulating in the fetoplacental blood flow depend on their regulation of potential targets [42]. Activation of the functional state of pro- and antioxidant systems, induced by OS and ROS suggests the launch of a cascade of sequential reactions by changing gene expression mediated by miRNA-dependent regulation. Taking into account the established correlations between the expression of miR-125b-5p and miR-451a, data on the assessment of microcirculation of the placenta vascular system and fetal cerebral hemodynamics, and differences in the expression of these miRNAs in newborns with NRDS and VLBW, we found it interesting to evaluate the participation of miR-125b-5p and miR-451a in signaling pathways. Using bioinformatics databases (MiRTarBase4.5, DAVID6.8, PANTHER14.1), we searched for target genes and determined a number of pathways, as shown in Figure 4.

We noted a significant number of common key signaling pathways for the studied miRNAs, among which the focus is on those associated with angiogenesis, hypoxia’s response via HIF activation (HIF-1 signaling pathway), various growth factors (PDGF signaling pathway, EGF receptor signaling pathway, FGF signaling pathway, TGF-β signaling pathway), differentiation and survival of neurons (neuro-trophin signaling pathway), and also with the genes involved. Moreover, the oxidative stress response, VEGF signaling pathway (regulation of vasculo-genesis and angiogenesis), platelet activation, GABA B receptor II signaling (inhibition of neurotransmitter release), and hematopoietic cell lineage were determined among the pathways specific for miR-125b-5p. The MAPK signaling pathway, which is one of the key pathways that activates proliferation of endothelial cells in angiogenesis and regulates the synaptic plasticity of neurons, the PI3K-Akt signaling pathway (phosphatidylinositol 3-kinase (PI3K) and protein kinase B (Akt)) associated with OS, and the TNF signaling pathway (regulation of immune cells) were determined to be specific for miR-451a. The obtained data demonstrate that the multidirectional effects of the expression of one miRNA can only be achieved by finely tuned regulation of genes in the context of signaling pathways. In general, it is important to emphasize that the present study allowed us to establish a relationship between the expression of oxidative stress-associated miRNAs in umbilical cord blood plasma, hemodynamic changes in the functional mother-placenta-fetus system, and perinatal outcomes.

## 5. Conclusions

Epigenetic miRNA-dependent modulation of the expression of genes responsible for redox homeostasis in the context of pregnancy complications may significantly contribute to adverse perinatal outcomes. However, to prevent long-term consequences associated with, in particular, cardiovascular diseases in children with growth restriction, further studies are needed to identify and validate marker miRNAs associated with OS and endothelial dysfunction in newborns at risk.

## Figures and Tables

**Table 1:** Clinical characteristics, ultra-sonography and Doppler sonography of pregnant women.

	<34 GA			>34 GA		
	Pregnant Women Cohort with FGR (Fetal Growth Restriction) (*n* = 9)	Control Groups (*n* = 13)	*p*-Value	Pregnant Women Cohort with FGR (*n* = 13)	Control Groups (*n* = 13)	*p*-Value
Gestational age at the time of delivery, weeks	30.5 ± 1.9	31.5 ± 1.9	0.24	37 (36.3–38.3)	38.2 (36.4–38.5)	0.31
Maternal age	33.2 ± 2.9	32 ± 4.8	0.49	32.4 (32–34)	30.67 (30.67–30.67)	0.007
Preeclampsia, *n* (%)	5 (55.6)	0 (0)	0.005	2 (15.4)	0 (0)	0.49
Ratio of placental dysfunction markers (sFLT-1/PGF; 1,5–7)	362.3 ± 297.6	NA	–	67 ± 31	NA	–
^‡^ Pulsatility index of Uterine Artery (PI UtA.) [18]	2.24 ± 1.25	−0.11 ± 0.44	<0.001	0.41 (0.11–1.31)	0.38 (0.18–0.42)	0.52
^‡^ Pulsatility index of Umbilical Artery (PI UA) [19]	3.12 ± 1.43	−0.28 ± 0.44	<0.001	0.75 (0.19 – 0.96)	0 (−0.12–0.19)	0.008
^‡^ Pulsatility index of Middle Cerebral Artery (PI MCA) [19]	−0.56 ± 0.72	−0.12 ± 0.4	0.12	−0.43 (−0.68–0.36)	0.57 (0.24–0.68)	0.05
Cerebral Placental Ratio (CPR > 1)	0.94 ± 0.16	2.33 ± 0.44	<0.001	1.45 (1–2.08)	2.11 (2.08–2.21)	0.02
Estimated Fetal Weight, ultra-sonography (EFW)	949 (922–1000)	1734 (1555–2160)	0.005	2136 (1835–2465)	3310.6 (3096–3548)	<0.001
Impaired fetoplacental and uteroplacental blood flow, *n* (%)	9 (100)	0 (0)	<0.001	7 (53.8)	0 (0)	0.003
IFC (indicator of fetal condition) (CTG - cardiotocography)	NA	NA	–	1.27 ± 0.94	0.47 ± 0.24	0.01
Increase of blood flow in MCA (PI below 5th centile), *n* (%)	4 (44.4)	0 (0)	0.01	2 (15.4)	0 (0)	0.49
Null end-diastolic blood flow or absence of blood flow in umbilical artery, *n* (%)	3 (33.3)	0 (0)	0.05	0 (0)	0 (0)	–
Oligohydramnios, *n* (%)	6 (66.6)	0 (0)	0.005	7 (53.8)	0 (0)	0.003

NA—Not analysed. GA—gestational age (weeks). For normal distribution, the mean value (M) and standard deviation (SD) in the M ± SD format were used. In case of non-normal distribution—the median (Me) and quartiles Q1, Q3 in the format Me (Q1–Q3) were used. ^‡^ The relative units normalized to the range of the reference values of PI (UtA, UA, MCA) are indicated (see 2.6. Statistical Data Analysis); Initial values see in Supplementary Table S1.

**Table 2:** Clinical state assessment of newborns with **FGR** and neonatal outcomes.

	<34 GA			>34 GA		
	Pregnant Women Cohort with FGR (*n* = 9)	Control Groups (*n* = 13)	*p*-Value	Pregnant Women Cohort with FGR (*n* = 13)	Control Groups (*n* = 13)	*p*-Value
BW, (grams)	997 ± 392	1825.3 ± 492	0.001	1996.8 ± 399.6	3113.5 ± 436.3	<0.001
Percentile, *n* (%):						
below 3d	3 (33.3)	–	–	9 (69.2)	–	–
below 10th	6 (66.6)	–	–	3 (23.1)	–	–
below 50th	–	3 (23)	–	–	2 (15.3)	–
above 50th	–	10 (76.9)	–	1 (7.7)	11 (84.6)	–
VLBW, *n* (%)	1 (11.1)	2 (15.4)	1	3 (23.1)	0 (0)	0.21
ELBW, *n* (%)	8 (88.9)	1 (7.7)	<0.001	–	–	–
APGAR 1 min	5 (4–6)	7 (6–7)	0.05	8 (7–8)	8 (8–8)	0.09
APGAR 5 min	7 (7–7)	7 (7–8)	0.19	8 (8–9)	9 (9–9)	0.02
Umbilical cord blood Ph (<7,2) [21]	7.3 ± 0.07	7.35 ± 0.1	0.13	7.32 (7.32–7.33)	7.37 (7.37–7.37)	0.001
Umbilical cord blood pO_2_, mm Hg (16−43) [22]	44.8 (15.7–58)	32.5 (29–37.5)	0.47	23.14 (23.14–23.14)	24 (24–24)	0.005
Umbilical cord blood pCO_2_, mm Hg (28−57) [22]	47.7 ± 11.2	39.5 ± 15.7	0.16	46.88 ± 4.84	38.35 ± 0.31	<0.001
Lactate umbilical cord blood (up to 2 mmol/L)	4.52 (3.9–4.52)	1.75 (1.6–1.75)	<0.001	NA	NA	–
Platelet level (151 − 320 × 10 × 9/L)	109 (100–152)	248 (201–268)	0.004	257.3 ± 83.2	281 ± 66.9	0.4
Neonatal Outcomes:						
Intrauterine pneumonia, *n* (%)	8 (88.8)	2 (15.4)	<0.002	3 (23.1)	0 (0)	0.21
NRDS, *n* (%)	3 (33.3)	0 (0)	0.05	0 (0)	0 (0)	–
Neonatal asphyxia	7 (77.7)	0 (0)	0.02	1 (7.7)	0 (0)	1
IVH, *n* (%)	2 (22.2)	0 (0)	0.16	3 (23.1)	0 (0)	0.21
Cerebral ischemia, *n* (%)	3 (33.3)	0 (0)	0.05	1 (7.7)	0 (0)	1
Anemia, *n* (%)	6 (66.7)	1 (7.7)	0.006	1 (7.7)	0 (0)	1
Neonatal hyper-bilirubinemia, *n* (%)	4 (44.4)	2 (15.4)	0.18	2 (15.4)	0 (0)	0.48
NEC, *n* (%)	2 (22.2)	0 (0)	0.1	0 (0)	0 (0)	–
Neuro-sonography:						
Subependymal hemorrhage, *n* (%)	4 (44.4)	0 (0)	0.02	1 (7.7)	0 (0)	1
Cerebral cyst, *n* (%)	2 (22.2)	0 (0)	0.16	3 (23.1)	0 (0)	0.21
Hyper-echogenicity of periventricular zone, *n* (%)	5 (55.6)	0 (0)	0.004	0 (0)	0 (0)	–
IVH, *n* (%)	2 (22.2)	0 (0)	0.16	3 (23.1)	0 (0)	0.21
Periventricular ischemia, *n* (%)	3 (33.3)	0 (0)	0.05	0 (0)	0 (0)	–

GA—gestational age (weeks). BW—birth weight. VLBW—very low birth weight. ELBW—extremely low birth weight. NRDS—neonatal respiratory distress syndrome. IVH—intraventricular hemorrhage. NEC—necrotizing enterocolitis. NA—Not analysed. For normal distribution, the mean value (M) and standard deviation (SD) in the M ± SD format were used. In case of non-normal distribution, the median (Me) and quartiles Q1, Q3 in the format Me (Q1–Q3) were used.

**Table 3:** The results of a correlation of miRNA expression level in cord blood with Doppler measurement, laboratory indicators of pregnant women and clinical state assessments of newborns with FGR.

		FGR			
		<34 GA		>34 GA	
miRNA (ΔCt)	Parameter	r *	*p* **	r *	*p* **
miR-125b-5p	PI UA	–	–	−0.54	0.03
	Platelet of pregnant	–	–	−0.51	0.005
	Weight of newborn	–	–	0.41	0.03
miR-451a	IFC (CTG)	–	–	−0.38	0.04
	PI UA	–	–	−0.59	0.001
	CPR	–	–	0.48	0.009
	Prothrombin index	–	–	−0.46	0.01

* r is a Spearman rank correlation coefficient; ** *p* is the statistical significance of correlation. GA—gestational age (weeks). PI UA—pulsatility index of umbilical artery. CPR—cerebral placental ratio. IFC—indicator of fetal condition.

**Table 4:** Results of the correlation of miRNA expression level in placental tissue with Doppler measurement of pregnant women and clinical state assessments of newborns with FGR.

		FGR			
		<34 GA		>34 GA	
miRNA (ΔCt)	Parameter	r *	*p* **	r *	*p* **
miR-125b-5p	EFW	0.54	0.05	–	–
	PI MCA	0.79	0.0007	–	–
	CPR	0.73	0.003	–	–
	Weight of newborn	0.56	0.04	–	–
miR-451a	PI UtA	0.62	0.01	–	–
	APGAR 1 min	–	–	−0.57	0.01

* r is a Spearman rank correlation coefficient; ** *p* is the statistical significance of correlation. GA—gestational age (weeks). EFW—estimated fetal weight (ultrasonography). PI MCA—pulsatility index of Middle Cerebral Artery. CPR—cerebral placental ratio. PI UtA—pulsatility index of Uterine Artery.

**Table 5:** Results of correlation of Doppler measurement of pregnant women and clinical state assessments of newborns with FGR.

		**FGR < 34 GA**							
		**Newborns**							
		**Birth Weight**		**APGAR** **1 min**		**APGAR** **5 min**		**Platelet**	
		r *	*p* **	r *	*p* **	r *	*p* **	r *	*p* **
**Doppler indices of pregnant women**	EFW	–	–	0.44	0.04	0.56	0.008	–	–
	IFC (CTG)	−0.64	0.001	−0.59	0.004	–	–	−0.49	0.02
	PI UtA	−0.63	0.002	−0.54	0.01	–	–	–	–
	PI UA	–	–	−0.44	0.04	−0.42	0.05	−0.67	0.0001
	PI MCA	–	–	–	–	–	–	–	–
	CPR	–	–	0.55	0.01	–	–	0.68	0.0001
		**FGR > 34 GA**							
		**Newborns**							
		**Birth Weight**		**APGAR** **1 min**		**APGAR** **5 min**		**Platelet**	
		r *	*p* **	r *	*p* **	r *	*p* **	r *	*p* **
	EFW	–	–	0.6	0.0001	0.68	0.0001	–	–
	IFC (CTG)	−0.48	0.01	–	–	–	–	−0.43	0.02
	PI UtA	−0.38	0.05	–	–	–	–	−0.38	0.04
	PI UA	−0.67	0.0001	–	–	−0.39	0.04	−0.39	0.03
	PI MCA	–	–	–	–	–	–	0.39	0.04
	CPR	0.5	0.006	–	–	–	–	–	–

* r is Spearman rank correlation coefficient; ** *p* is the statistical significance of correlation. GA—gestational age (weeks). EFW—estimated fetal weight (ultrasonography). IFC (CTG)—indicator of fetal condition (cardiotocography). PI UtA—pulsatility index of Uterine Artery. PI UA—pulsatility index of umbilical artery. PI MCA—pulsatility index of Middle Cerebral Artery. CPR—cerebral placental ratio.

## Supplementary Materials

The following are available online at https://www.mdpi.com/2077-0383/9/10/3227/s1, Table S1: Initial values of pulsatility indexes of pregnant.
